# Chitosan Cross-Linking with Acetaldehyde Acetals

**DOI:** 10.3390/biomimetics7010010

**Published:** 2022-01-06

**Authors:** Alexander Pestov, Yuliya Privar, Arseny Slobodyuk, Andrey Boroda, Svetlana Bratskaya

**Affiliations:** 1 I. Ya. Postovsky Institute of Organic Synthesis, Ural Branch of the Russian Academy of Sciences, 22, S. Kovalevskoy Str., 620990 Yekaterinburg, Russia; 2 Institute of Chemistry Far Eastern Branch, Russian Academy of Sciences, 159, Prosp. 100-letiya Vladivostoka, 690022 Vladivostok, Russia; privar.juliya@gmail.com (Y.P.); ampy@ich.dvo.ru (A.S.); 3 A.V. Zhirmunsky National Scientific Center of Marine Biology, Far Eastern Branch of Russian Academy of Sciences, 17, Palchevskogo Street, 690041 Vladivostok, Russia; borodandy@gmail.com

**Keywords:** chitosan, acetaldehyde acetals, hydrogel, rheology

## Abstract

Here we demonstrate the possibility of using acyclic diethylacetal of acetaldehyde (ADA) with low cytotoxicity for the fabrication of hydrogels via Schiff bases formation between chitosan and acetaldehyde generated in situ from acetals in chitosan acetate solution. This approach is more convenient than a direct reaction between chitosan and acetaldehyde due to the better commercial availability and higher boiling point of the acetals. Rheological data confirmed the formation of intermolecular bonds in chitosan solution after the addition of acetaldehyde diethyl acetal at an equimolar NH_2_: acetal ratio. The chemical structure of the reaction products was determined using elemental analysis and ^13^C NMR and FT-IR spectroscopy. The formed chitosan-acetylimine underwent further irreversible redox transformations yielding a mechanically stable hydrogel insoluble in a broad pH range. The reported reaction is an example of when an inappropriate selection of acid type for chitosan dissolution prevents hydrogel formation.

## 1. Introduction

Expanding directions of chitosan application to obtain new organic materials requires the development of versatile approaches for this aminopolysaccharide modification. This modification must be targeted not only toward the introduction of ionic functional groups [1] to broaden the pH window of the polymer solubility, but also to cross-link macromolecules to limit the solubility in acidic media [2]. The latter type of modification allows the fabrication of chitosan hydrogels for pharmaceutical and medical use with tunable swelling, which can be controlled via the type and density of the cross-linking [3,4].

Taking into account that chitosan is a polyamine, the most effective reagents for cross-linking are dialdehydes [2,5,6], which ensure rapid formation of imine in an aqueous medium under mild conditions [7,8]. Commercial glutaric dialdehyde is often used to crosslink chitosan [2,9]. However, its natural toxicity limits its use for biomedical applications [10,11]. To solve this problem, recent studies suggest using the products of the partial oxidation of polysaccharides as cross-linkers [5,12,13,14,15,16,17,18,19] and their functional derivatives [20,21]. In this case, the natural origin and high molecular weight of the cross-linkers, as well as the irreversibility of the formed intermolecular bonds, eliminate the disadvantages of glutaric dialdehyde. However, non-selective methods of cross-linker synthesis and the irregular structure of carbohydrate polyaldehydes lead to the low reproducibility of this fabrication method and of resultant hydrogels’ properties.

Despite their monofunctionality, aromatic aldehydes are capable of yielding chitosan hydrogels stabilized via non-covalent interactions without the formation of cross-linkages [22,23]. In many cases, reactions between chitosan and aldehydes result in the formation of several products and but the mechanism of gelation, which depends on the aldehyde structure, still remains a subject of investigation [24,25,26,27,28].

Among monoaldehydes, acetic and glycerol aldehydes have potential advantages for biomedical use, since they are natural metabolites of biochemical reactions. However, only a few reports on the application of these reagents are available [25,29,30]. Possible obstacles are their low commercial availability, and, in the case of acetaldehyde, its low boiling point. The use of acetals of these aldehydes may eliminate the mentioned preparative difficulties and is worth investigating as an approach for hydrogel fabrication.

Thus, in order to evaluate the applicability of acetaldehyde acetals as crosslinking reagents for polyamines, in this work we have compared the reactivity of acetaldehyde diethyl acetal (ADA), paraldehyde, and 2-methyldioxane-1,3 with respect to chitosan.

## 2. Materials and Methods

### 2.1. Materials

Low molecular weight (CH-LMW) and high molecular weight (CH-HMW) chitosans were purchased from BioLog Heppe GmbH (Landsberg, Germany). The degree of acetylation (DA) was determined by ^1^H NMR spectroscopy to be 0.9 and 0.84 for CH-LMW and CH-HMW, respectively. The viscosity-average molecular weights of CH-LMW and CH-HMW were 30 kDa and 300 kDa, respectively. Acetaldehyde diethyl acetal (99% purity), paraldehyde (98% purity), and 2-methyldioxane-1,3 (97% purity) were purchased from Sigma-Aldrich (St. Louis, MO, USA).

### 2.2. Fabrication of Hydrogels

The hydrogels were obtained via the addition of acetals to 3% CH-HMW solution in acetic acid (equimolar ratio NH_2_: AcOH, pH 4.5) at molar ratios of NH_2_: acetal from 1:0.1 to 1:1 (see Table 1 for details) under intensive stirring at 25 °C. In a separate experiment, chitosan was dissolved in HCl solution at an equimolar NH_2_: HCl ratio, and the pH was adjusted to 4.5.

After gelation for 72 h, the reaction mass was treated with an aqueous NaOH solution, the precipitate was filtered off, washed to neutral reaction, and dried at room temperature to constant weight.

### 2.3. Characterization of Hydrogels

#### 2.3.1. Elemental Analysis, ^13^C NMR and FT-IR Spectroscopy

CHN contents in the reaction products (hydrogels), thoroughly washed with ethanol and dried in the vacuum oven, were determined in triplicates using a PE2400 CHNS analyzer (Perkin Elmer, USA); the degree of modification (DM) was calculated using the following formula:

DM=CNhydrogel−CNchit2

where *C/N_hydrogel_* and *C/N_chit_* are the atomic carbon/nitrogen ratios in hydrogels and chitosan, respectively; 2 is the number of carbon atoms in acetaldehyde.

The solid state ^13^C NMR spectra were recorded using the methods of cross polarization and spinning at the magic angle (CP/MAS) on a Bruker AVANCE AV-300 spectrometer, with a rotor diameter of 4 mm and at a spinning speed of 10 kHz. The chemical shifts were referenced to tetramethylsilane (TMS).Fourier transform infrared (FTIR) spectra were recorded using an IR Affinity-1 spectrometer with a MIRacle 10 FTIR accessory (Shimadzu, Kyoto, Japan).

#### 2.3.2. Rheological Properties

The rheological properties of the hydrogels formed 72 h after ADA addition to 3% CH-LMW and CH-HMW solutions in acetic acid at molar ratio NH_2_: acetal 1:1 were investigated by recording frequency sweeps in the range between 0.2 and 100 Hz at a temperature of 25 °C and a constant strain of 5% using a Physica MCR 301 rheometer (Anton Paar GmbH, Graz, Austria) with a plate–plate measuring system of a diameter of 25 mm.

#### 2.3.3. Hydrogels Swelling and Stability

Hydrogel chemical (hydrolytic) stability was investigated at 25 °C in PBS buffer (PanEco Ldt., Moscow, Russia); the pH in the acidic and basic range was adjusted with H_3_PO_4_ and NaOH, respectively. Colloid titration of supernatants was used to determine the content of the polymer, which was released from the hydrogels due to the hydrolysis of the cross-links, as described in [31]. The experiments were performed as follows: 300 mg of the hydrogel was immersed in 15 mL of PBS solution with adjusted pH value and gently agitated for 24 h using a Biosan PSU-20i orbital shaker (Latvia) at 30 rpm. Subsequently, an aliquot of the supernatant was taken for colloid titration with 0.001 mol/L standard solutions of sodium polyethylene sulphonate (PES-Na) at pH 2.5.

The hydrogel swelling was determined from the difference of in weight of the original and swollen hydrogels after 24 h.

#### 2.3.4. Cytotoxicity Study

The HCT116 cell line (Sigma-Aldrich Corp., St. Louis, MO, USA) was seeded at a density of 100 × 10^3^ cells/well in 1 mL of Dulbecco’s Modified Eagle’s Medium (DMEM, #12800017, Gibco™, Thermo Fisher Scientific, Altrincham, UK) supplemented with 10% (*v*/*v*) fetal bovine serum (FBS, HyClone, Logan, UT, USA), 3.7 mg/mL sodium bicarbonate (Sigma-Aldrich), 1x mixture of non-essential amino acids (MEM NEAA, Gibco), 100 U/mL penicillin (Gibco), and 100 µg/mL streptomycin (Gibco). Poly(ethylene glycol) diglycidyl ether, average Mn 500, CAS number 26403-72-5 (PEG DGE); acetaldehyde diethyl acetal (ADA); and salicylaldehyde (SA) were added to the wells at concentrations of 23 g/L, 21 g/L, and 4.4 g/L, respectively. The samples were cultivated at +37 °C, 5% CO_2_, and 90% relative humidity for 3.5 h. Than the cells were stained with 10 µM 2′,7′-dichlorodihydrofluorescein diacetate to assess the mitochondrial activity, 1 µM TO-PRO-3™ to detect apoptotic cells, and 1 µg/mL DAPI to discover dead cells. The data are presented as a percentage of intact control cells. Flow cytometric analyses were conducted after staining using a CytoFLEX flow cytometer (Beckman-Coulter, Brea, CA, USA) connected to a computer running CytExpert software (version 2.4, Beckman-Coulter). A detailed description of cell cultivation and flow cytometrical analysis is given in [31].

## 3. Results

### 3.1. Interaction of Chitosan with Acetaldehyde Acetals

**Figure 1:**
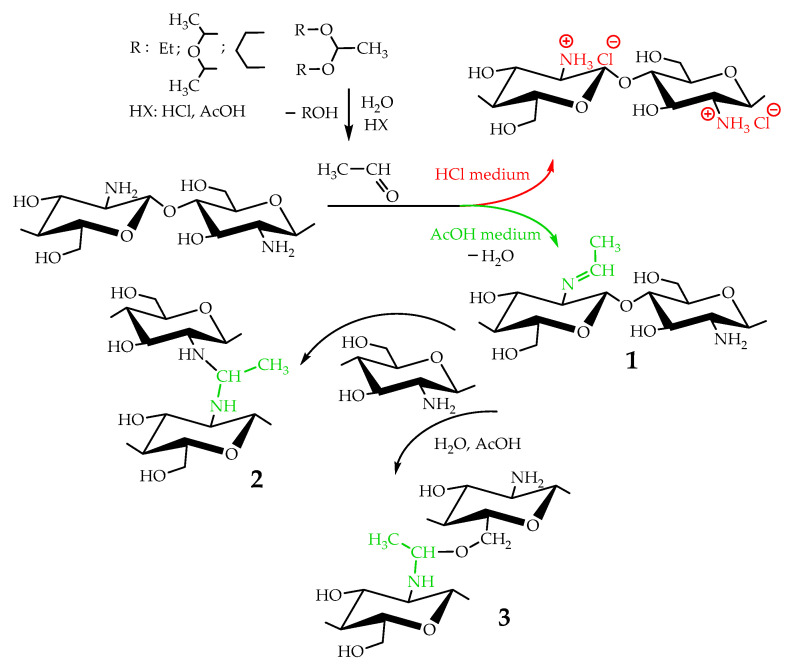
Scheme of interaction of chitosan with acetaldehyde acetals and possible further conversion of Schiff base.

Taking into account that the reaction of nucleophilic addition and the elimination of amines to the carbonyl group and the hydrolysis of acetals are catalyzed by Lewis acids [32], the reaction between chitosan and acetaldehyde diethyl acetal (ADA) was initially carried out in hydrochloric acid solution (Figure 1). However, the gelation of chitosan was not observed up to an equimolar chitosan/ADA ratio, and an elemental analysis showed that imine was not formed (Table 1). Although ADA hydrolyzes in HCl solution yielding acetaldehyde (Figure 1), protonation of the amino groups of chitosan with the strong acid levels their nucleophilicity and reduces reactivity in the addition reactions.

Using weak acetic acid as a reaction medium facilitates the addition of chitosan to acetaldehyde, so Schiff bases can be formed at low ADA/chitosan molar ratios (Table 1). Thus, despite the high degree of chitosan protonation in the reaction media (pH 4.5) sufficient for complete dissolution, weak organic acid did not affect nucleophilicity to such an extent as hydrochloric acid and the nucleophilic addition reaction proceeded (Figure 1). A similar phenomenon was observed earlier in the case of the Michael reaction—the nucleophilic addition of chitosan to acrylic acid [33].

As follows from the elemental analysis data summarized in Table 1, acetaldehyde generated in situ from ADA reacts with chitosan efficiently yielding products with a degree of modification (DM) up to 0.53. Cyclic acetals of acetaldehyde are more stable under the same conditions and do not react with chitosan, so no gelation or color change was observed in the solution for at least 5 days of observation. However, the synthetic potential of preparatively convenient cyclic acetals is worth further investigation.

**Figure 2:**
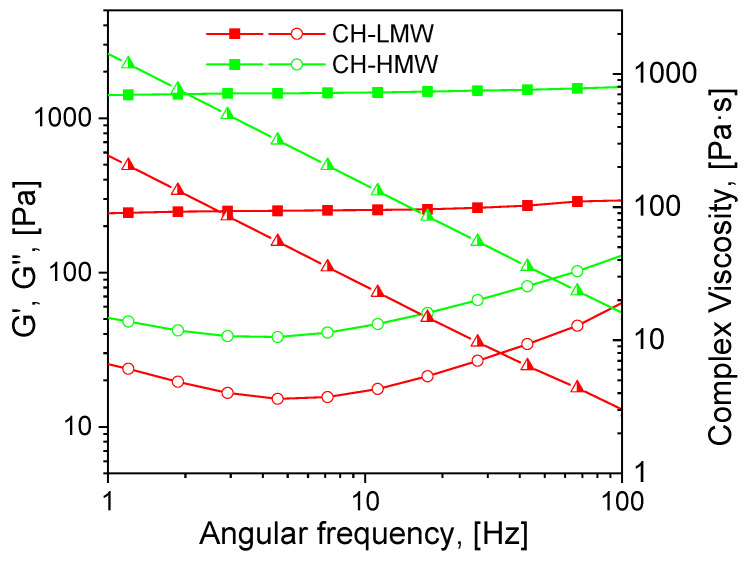
Mechanical spectra of low and high molecular weight chitosans (CH-LMW and CH-HMW, respectively) 72 h after addition of acetaldehyde diethyl acetal (ADA) at equimolar ADA:NH_2_ ratio. Squares—storage modulus (G′), circles—loss modulus (G″), triangles—complex viscosity.

Mechanical spectra recorded 72 h after the addition of acetaldehyde diethyl acetal (ADA) to the chitosan solutions revealed significant differences in the rheological properties of hydrogels depending on polymer molecular weight (Figure 2). The relatively low storage modulus of the hydrogels formed with both CH-LMW and CH-HMW suggests that grafts (Structure 1 in Figure 1) have significant contribution to the DM value, at least at high ADA/chitosan molar ratios. However, in comparison with propionaldehyde and n-butrylaldehyde, which yield chitosan Schiff bases with DM of 0.97–1.0 at tenfold molar excess [34], ADA has higher reactivity (Table 1). The storage modulus of the hydrogels formed with CH-HMW was above 1 kPa being in the range typical for hydrogel fabricated using other cross-linking agents for biomedical applications.

### 3.2. Analysis of Chemical Structure of the Hydrogels

**Figure 3:**
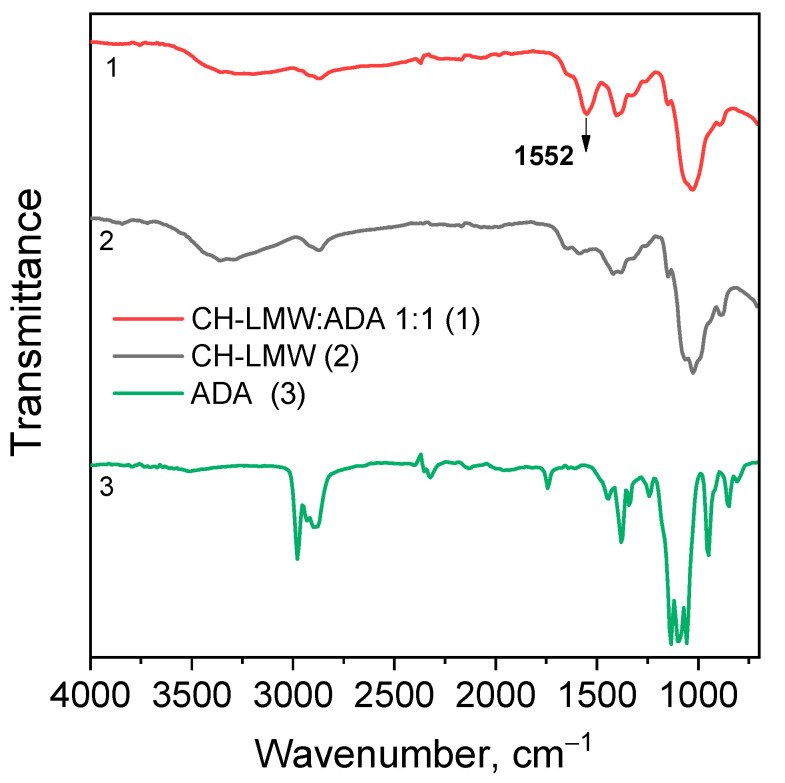
FT-IR spectra of chitosan cross-linked with acetaldehyde diethyl acetal (ADA) at molar ratio 1:1.

**Figure 4:**
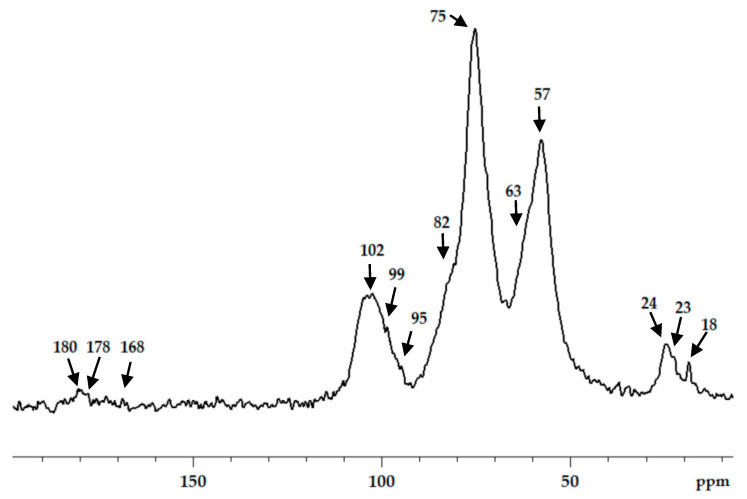
^13^C CP/MAS NMR spectra of chitosan cross-linked with acetaldehyde diethyl acetal (ADA) at molar ratio 1:1.

Chitosan interaction with ADA was proved by the emergence of a new imine band at 1552 cm^−1^ in the FT-IR spectrum (Figure 3). In the ^13^C NMR spectrum (Figure 4), the corresponding signals of the imino carbon atom at 168 ppm and methyl group at 18 ppm were observed but with low intensity. Taking into account elemental analysis data and calculated DM values, we assumed that the formed aliphatic imine (Structure 1 in Figure 1) was more reactive than aromatic salicylimine [28] and underwent further conversion via the reaction of nucleophilic addition with non-functionalized amino groups, as was observed for the reaction between chitosan and formaldehyde [24], or with hydroxyl groups [34].

**Figure 5:**
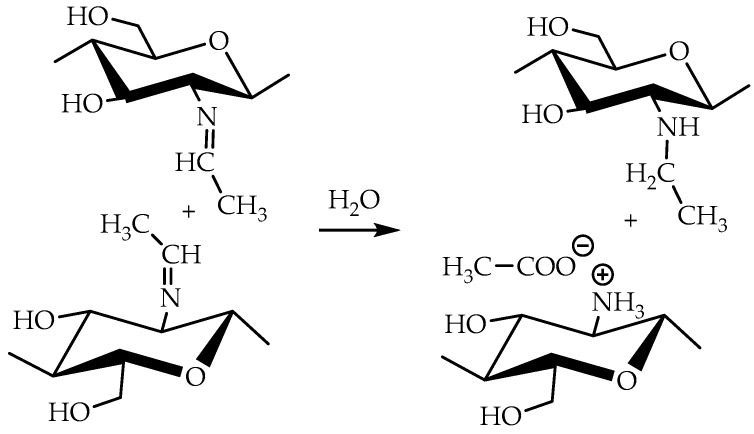
Possible conversion of Schiff bases of chitosan with acetaldehyde according to Cannizzaro reaction.

These reactions decrease the number of imino groups and led to chitosan crosslinking via diaminomethane (Structure 2 in Figure 1) or alkoxyaminomethane (Structure 3 in Figure 1) linkers. Indeed, in the ^13^C NMR spectrum (Figure 4) signals of acetal carbon atoms (95–99 ppm) were shifted to a stronger field relative to the signal of carbon in an acetal group (102 ppm) that indicates the connection of carbon to the less electronegative nitrogen atom (Figure 1). Another possible side process decreasing the number of imino groups is oxidation–reduction according to the Cannizzaro reaction [35] (Figure 5). Indeed, the ^13^C NMR spectrum (Figure 4) contains signals of acetate (23 and 180 ppm). According to Hartree–Fock quantum-chemical calculations with a standard basis 6-31G and Dalton software [36], the signal of carbon in a N-C-N motive (Structure 1, Figure 1) is expected at 60 ppm and can overlap with other signals typical for chitosan (Figure 4).

The aldehyde groups can also react with the hydroxyl groups of polysaccharides and polyols, leading to the formation of hemiacetals or acetals [14,16,37]. Formation of acetals in the reaction between ADA and chitosan cannot be proved by FT-IR spectroscopy due to the presence of an O−C−O structural motive in polysaccharides and overlapping corresponding band in the region 1000−1150 cm^−1^ with expected bands of newly formed acetals and hemiacetals. In the ^13^C NMR spectrum, chemical shifts of hemiacetal and acetal groups were expected at 94 and 108 ppm [38] but only minor contents of such fragments could be assumed (Figure 4).

**Figure 6:**
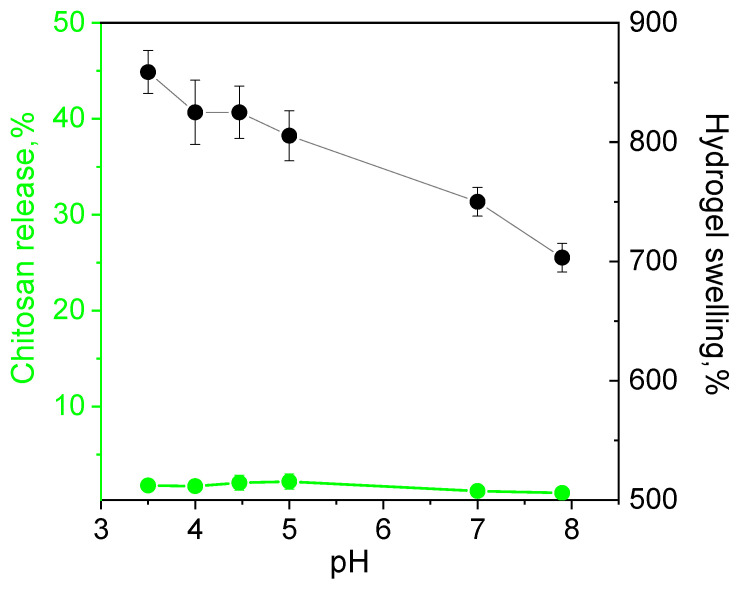
Dependence of hydrolytic stability (chitosan release) and swelling of hydrogels fabricated at equimolar ratios of chitosan and acetaldehyde dimethyl acetal (ADA) on pH.

The release of the polymer from the hydrogel fabricated at equimolar ratios of ADA with chitosan in pH range from 3 to 8 was below 1% indicating irreversibility of the cross-linking and high hydrolytic stability of the material even in acidic media. The swelling degree decreased slowly with the pH increase due to the lower protonation degree of chitosan (Figure 6).

**Figure 7:**
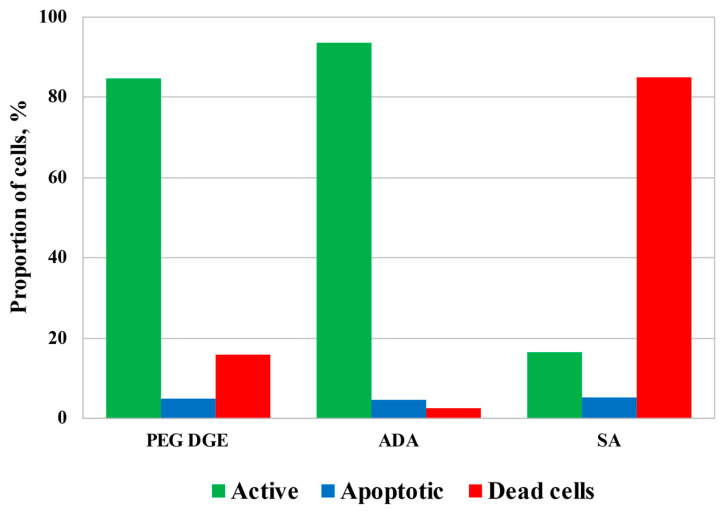
The results of flow cytometrical analysis of human colon carcinoma cells (HCT 116) cultivated for 3.5 h in the presence of cross-linking agents: poly(ethylene glycol) diglycidyl ether, average Mn 500, CAS number 26403-72-5 (PEG DGE), 23 g/L; acetaldehyde diethyl acetal (ADA), 21 g/L; salicylaldehyde (SA), 4.4 g/L.

Although a relatively high concentration of ADA was required for the fabrication of the mechanically stable hydrogels, Figure 7 demonstrates that ADA cytotoxicity was comparable with that of diglycidyl ether of polyethyleneglycol (PEG DGE), which is known as a highly biocompatible cross-linker [39,40], and was remarkably lower in comparison with salicylaldehyde (SA), selected as an example of monoaldehyde applicable for chitosan-based hydrogel fabrication [26,28]. Incubation of HCT116 cells for 3.5 h with cross-linkers at concentrations suitable for the fabrication of the hydrogels resulted in a slight decrease in mitochondrial activity to 85–93% (in the case of PEG DGE and ADA) and a consequent increase in the dead cell fraction. SA possessed the highest cytotoxicity leading to the death of about 85% of cells. The level of apoptosis remained almost unchanged in all tests indicating that cell death could result from an increase in cellular membrane permeability by cross-linkers.

## 4. Conclusions

The present work demonstrates the possibility of using acyclic acetals of acetaldehyde for the fabrication of chitosan-based hydrogels. Acetaldehyde generated in situ from acetaldehyde diethyl acetal (ADA) is capable, despite its monofunctional character, of reacting with acetate chitosan and forming new covalent intermolecular bonds of the diaminomethane or alkoxyaminomethane type. However, the reaction does not proceed if chitosan is dissolved in hydrochloric acid. This is another example of an inappropriate selection of acid type preventing hydrogel formation. In earlier reports by us, for example [40], chitosan hydrogel fabrication using diglycidylethers of glycols as cross-linkers in acidic media was feasible only if chitosan was dissolved in hydrochloric acid. The mechanism of chitosan cross-linking with acetaldehyde is based on the conversion of initially formed imino groups via the reaction of nucleophilic addition of amino or hydroxyl groups of unmodified chitosan units. The fabricated hydrogels were insoluble over a wide pH range. The cytotoxicity of ADA was very low, and comparable with that of poly(ethylene glycol) diglycidyl ether used in biofabrication as a highly biocompatible cross-linker.

## Figures and Tables

**Table 1:** Elemental composition and degree of modification (DM) of high molecular weight chitosan with derivatives of acetaldehyde.

Acetal	Acid	NH2: Acetal Molar Ratio	Content, Weight %		DM
			C	N	
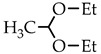 ADA	HCl	1:0.5	41.46	7.49	0
	AcOH	1:1	44.74	6.96	0.53
		1:0.5	45.20	7.71	0.20
		1:0.25	44.92	7.72	0.17
		1:0.1	44.74	7.90	0.08
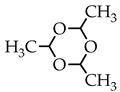 paralaldehyde	AcOH	1:0.17	42.68	7.69	0
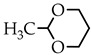 2-methyldioxane-1,3	AcOH	1:1	41.58	7.53	0

## Data Availability

Data are available from the authors upon request.
